# CSF formation rate—a potential glymphatic flow parameter in hydrocephalus?

**DOI:** 10.1186/s12987-024-00560-6

**Published:** 2024-07-10

**Authors:** Sara Qvarlander, Nina Sundström, Jan Malm, Anders Eklund

**Affiliations:** 1 Department of Diagnostics and Intervention, Biomedical Engineering and Radiation Physics, Umeå University, Umeå, Sweden; 2 Department of Clinical Science, Neurosciences, Umeå University, Umeå, Sweden

**Keywords:** Glymphatic system, Idiopathic normal pressure hydrocephalus, CSF dynamics, CSF production

## Abstract

**Background:**

Studies indicate that brain clearance via the glymphatic system is impaired in idiopathic normal pressure hydrocephalus (INPH). This has been suggested to result from reduced cerebrospinal fluid (CSF) turnover, which could be caused by a reduced CSF formation rate. The aim of this study was to determine the formation rate of CSF in a cohort of patients investigated for INPH and compare this to a historical control cohort.

**Methods:**

CSF formation rate was estimated in 135 (75 ± 6 years old, 64/71 men/women) patients undergoing investigation for INPH. A semiautomatic CSF infusion investigation (via lumbar puncture) was performed. CSF formation rate was assessed by downregulating and steadily maintaining CSF pressure at a zero level. During the last 10 min, the required outflow to maintain zero pressure, i.e., CSF formation rate, was continuously measured. The values were compared to those of a historical reference cohort from a study by Ekstedt in 1978.

**Results:**

Mean CSF formation rate was 0.45 ± 0.15 ml/min (N = 135), equivalent to 27 ± 9 ml/hour. There was no difference in the mean (p = 0.362) or variance (p = 0.498) of CSF formation rate between the subjects that were diagnosed as INPH (N = 86) and those who were not (N = 43). The CSF formation rate in INPH was statistically higher than in the reference cohort (0.46 ± 0.15 vs. 0.40 ± 0.08 ml/min, p = 0.005), but the small difference was probably not physiologically relevant. There was no correlation between CSF formation rate and baseline CSF pressure (r = 0.136, p = 0.115, N = 135) or age (-0.02, p = 0.803, N = 135).

**Conclusions:**

The average CSF formation rate in INPH was not decreased compared to the healthy reference cohort, which does not support reduced CSF turnover. This emphasizes the need to further investigate the source and routes of the flow in the glymphatic system and the cause of the suggested impaired glymphatic clearance in INPH.

## Background

Idiopathic normal pressure hydrocephalus (INPH) is a neurodegenerative disease characterized by gait/balance disturbance, enlarged ventricles and disturbance of the cerebrospinal fluid (CSF) circulation. The glymphatic system was recently suggested as a system for clearance of metabolic products from the brain, where metabolites are cleared by flow of CSF through the parenchyma [1]. Glymphatic MR imaging with intrathecal injection of a contrast agent (gadolinium) has indicated delayed clearance of contrast from the brain and CSF system in INPH [2–5]. These findings suggest a reduced CSF turnover, which could be explained by a reduced CSF formation rate. Furthermore, a small study has suggested that the formation rate is reduced in chronic hydrocephalus [6]. Animal studies have also suggested that using acetazolamide to inhibit CSF formation rate can reduce glymphatic clearance [7], and reduced formation rate with aging [8] has been suggested as a potential mechanism contributing to impaired glymphatic function in dementia [9, 10]. Altogether, this implies that reduced CSF formation rate may be an important pathophysiological factor in INPH.

Data on CSF formation rate in humans is scarce, and the reliable methods are highly invasive and therefore difficult to perform in healthy subjects. One of the few available reliable reference materials for CSF formation rate in subjects without neurological or circulatory disorder is a cohort from Ekstedt using a method based on CSF withdrawal at a low constant pressure [11]. That data opens for comparing CSF formation rate in INPH and healthy controls, to uncover potential abnormalities. The aim of this study was to determine the formation rate of CSF in 137 patients investigated for INPH and compare this to the previous reported cohort of reference subjects.

## Methods

### Subjects

Patients with communicating hydrocephalus on CT or MRI are referred from local hospitals to our tertiary hydrocephalus center if they have corresponding hydrocephalus symptoms. All patients are investigated with a standardized protocol. It includes case history, neurological examination, blood and cerebrospinal fluid samples, MRI of the brain, short-term tap test, cerebrospinal fluid dynamics investigation and in doubtful cases an external lumbar drainage for three days.

For the present study, subjects were included if they had undergone a CSF dynamic investigation (CELDA system, Likvor AB, Umeå, Sweden) that included estimation of CSF formation rate (Q_f_) by drainage of fluid at zero CSF pressure. The study population consisted of 137 subjects. Since CSF formation rate per definition is positive (a negative value would imply absorption), estimated values below zero were considered measurement error and excluded from analysis (N = 2). The final population thus consisted of 135 subjects; the clinical characteristics of the population are presented in Table 1. The subjects were categorized into three groups based on their final diagnosis (INPH, secondary hydrocephalus (SH) or ventriculomegaly (VM)). Definitions of the diagnostic groups are described in the Appendix.

**Table 1:** Characteristics of the study population

Subjects	All patients	INPH	SH	VM
N (% of total)	135	86 (64%)	6 (4%)	43 (32%)
Age (mean ± SD (range))	75 ± 6 (61–86) years	74 ± 6 (61–85) years	72 ± 6 (61–77) years	77 ± 6 (63–86) years
Men/Women (%)	64 (47%)/71 (53%)	41 (47%)/47 (53%)	5 (83%)/1 (17%)	19 (44%) / 24 (56%)
P_baseline_ (mean ± SD)	10.5 ± 2.5 mmHg	10.9 ± 2.6 mmHg	10.0 ± 2.1 mmHg	9.7 ± 2.3 mmHg

*INPH* Idiopathic normal pressure hydrocephalus, *SH* Secondary hydrocephalus, *VM * Ventriculomegaly (see Appendix for definitions)

### CSF infusion investigation

**Fig. 1:**
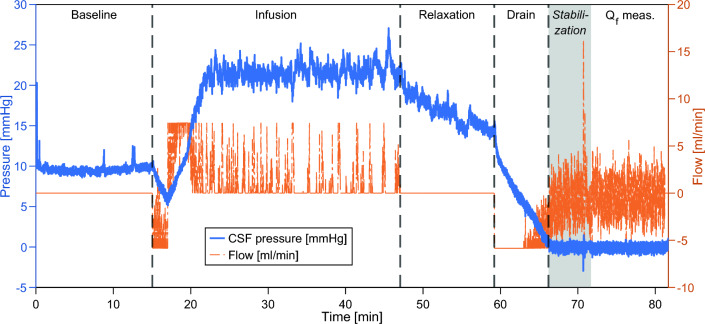
The graph shows pressure (blue solid line) and flow (orange dashed line) during a CSF infusion investigation. Note that in the graph fluid withdrawal from the patient is defined as negative flow, by convention, i.e., for the Q_f_ measurement a negative average flow corresponds to a positive Q_f_ estimate. The CSF infusion investigation included baseline pressure (P_baseline_), an infusion section with outflow resistance assessment (the design of this section varied during the study), pressure relaxation and, at the end, a tap-test (drain). During the tap test CSF was actively withdrawn using the peristaltic two-way pump, to lower the pressure to zero and then keep it there for 15 min or until the total volume withdrawn equaled 50 ml. The dashed vertical lines illustrate the start/end of the different parts of the investigation; the gray area of the graph shows the stabilization period when the pressure first reached zero that was excluded from the CSF formation rate (Q_f_) measurement

The CSF formation rate was assessed using a standardized CSF dynamic investigation (Fig. 1) using a dedicated infusion device (CELDA, Likvor AB, Umeå, Sweden). In brief, the device measured pressure with a fluid-catheter system via lumbar puncture; one needle/catheter was used for pressure measurement and another for infusion/withdrawal. Pressure was sampled at 100 Hz and averaged to 1 Hz. The sensors were zeroed to atmospheric pressure with the reference level at the external auditory canal; all pressures here reported are thus in relation to atmospheric pressure. All measurements were performed with the patient in the supine position, where there is excellent agreement between pressure levels measured with this method and those measured by an intracranial pressure sensor [12].

After baseline CSF pressure measurement and infusion to determine CSF outflow resistance, CSF was withdrawn to perform a tap test; part of the tap test withdrawal was used to estimate CSF formation rate (Q_f_). The measurement protocol was pressure-controlled, meaning that the peristaltic pump of the infusion device could infuse and withdraw CSF to maintain a preset CSF pressure level (regulation frequency 1 Hz). For the tap test, the scheme was to reduce the CSF pressure to zero, a pressure where there should not be any absorption along venous or lymphatic pathways. By simply measuring the withdrawal rate needed to then maintain the zero pressure, we can estimate the CSF formation rate, since we should then withdraw as much CSF as is formed. The target pressure for regulation was thus set to zero and when this pressure was reached, it was regulated at this level for 15 min. The detection of when the 15 min began was based on when pressure first fell within ± 0.4 mmHg (a single value in this range was required), serving as an automatic trigger for the timing. At this point the pressure had not necessarily reached a stable zero level. An initial pressure stabilization period was therefore always excluded from the analysis. Based on visual inspection of all pressure measurements, four minutes was deemed an appropriate length for this stabilization, to ensure most cases had achieved a stable pressure before the actual Q_f_ measurement began. For consistency between subjects, the estimation of Q_f_ was always based on 10 min of data with regulation to zero pressure (after a minimum four minutes of stabilization). Thus, at least 14 min of measurement after the automatic trigger was an inclusion criterion for the study. Accordingly, subjects that had measurements that were shorter than this, due to the desired tap test volume (40–50 ml) being reached (N = 77), technical issues withdrawing CSF with the pump (N = 10), patient discomfort, e.g., tap test related headache (N = 44), or an older shorter protocol (N = 14) were not included in the study (numbers estimated based on available investigational notes).

### Theory and calculation

As described, the formation rate of CSF was measured by estimating the average withdrawal rate needed to maintain CSF pressure at zero, i.e., below the venous and lymphatic pressure. The approach furthermore assumes that absorption of CSF can be considered as a pressure driven unidirectional flow. I.e., that there is no backflow through the arachnoid granulations if the CSF pressure is lower than pressure in the dural venous sinuses. We also assume that pressure in the dural venous sinuses, expected to be in the range of 6–9 mmHg, is higher than zero (i.e. our CSF pressure level) during the investigation (with the patient supine) [13–15]. The same must hold for lymphatic pressure [15, 16], i.e., no absorption occurs to lymph when CSF pressure is at zero. This means that the CSF withdrawn by the infusion device corresponds to all the CSF that is formed during the same period, i.e. the withdrawal rate corresponds to the CSF formation rate. To interpret this as the CSF formation rate during normal conditions, i.e., at resting pressure, one must assume that formation rate of CSF is independent of intracranial pressure in the range from normal resting pressure down to zero. This is supported by the current understanding of CSF formation resulting from active secretion of fluid, rather than as from ultrafiltration of plasma [17].

To average out CSF pressure variation due to cardiac cycle, respiratory cycle and vasomotion with autoregulatory origin the estimation of formation rate was based on a minimum of ten minutes. This was chosen to be the smallest feasible period to achieve a stable estimate. Q_f_ was thus defined as mean pump flow during the last 10 min of zero pressure regulation. Baseline pressure (P_baseline_) was defined as the average CSF pressure during the last 5 min of the baseline measurement. To estimate the uncertainty of the Q_f_ measurements, we determined a standard error of each Q_f_ estimate based on dividing the measurement period into two 5-min halves. I.e., the flow was averaged for each half and the standard deviation of these two values was then calculated and divided by the square root of 2, to derive a standard error for the 10-min mean based on two 5-min estimates.

### The reference cohort and method

This study was motivated by the published healthy reference cohort for CSF formation rate from Ekstedt’s 1978 study [11]. The cohort consists of 58 subjects (31 women and 27 men) “whose medical history, medical and neurological investigation, and follow-up for at least two years, made it highly probable that they had no organic neurological or circulatory disorder.”[11] The age range of that cohort was 18–82 years; based on their graphical representation of the age distribution, we estimated that 21 of their subjects were within the age range of our study.

The method used in the Ekstedt study is very similar to ours, assessing average drainage rate at a low constant pressure [11]. They achieved pressure-controlled drainage using a bottle with artificial CSF connected to one of the needles, with the bottle placed at the appropriate height to transmit a constant external pressure to the CSF system. In their case, the pressure level was 0.25 kPa (1.9 mmHg), i.e., somewhat higher than ours. They also allowed for a stabilization period, but then measured for a longer period than in the present study, 20–30 min.

### Statistics

While the hydrocephalus patients were divided into three groups, the secondary hydrocephalus group only included six patients, therefore, statistical comparisons between different hydrocephalus diagnoses were only performed for the INPH and VM groups. Q_f_ in these two groups, as well as in men and women, were compared using independent samples t-tests, (assuming equal variances), and Levene’s test for equality of variances. Correlation between Q_f_ and age, as well as Q_f_ and baseline CSF pressure (P_baseline_), were analyzed using Pearson’s correlation coefficient. These correlations were also analyzed using partial linear correlation, correcting for age or P_baseline_, respectively. These statistical tests were performed using PSAW Statistics (18.0.3, IBM, Armonk, NY, USA). For the historical reference cohort no individual data was available, therefore, to compare this cohort with the group of patients with INPH, tests based on mean, standard deviation (SD) and samples sizes were implemented in an in-house MATLAB script (R2023b, The MathWorks, Inc, Natick, MA, USA). A Welch’s unequal variances t-test was used for comparing the means and an F-test for comparing the SD.

## Results

**Fig. 2:**
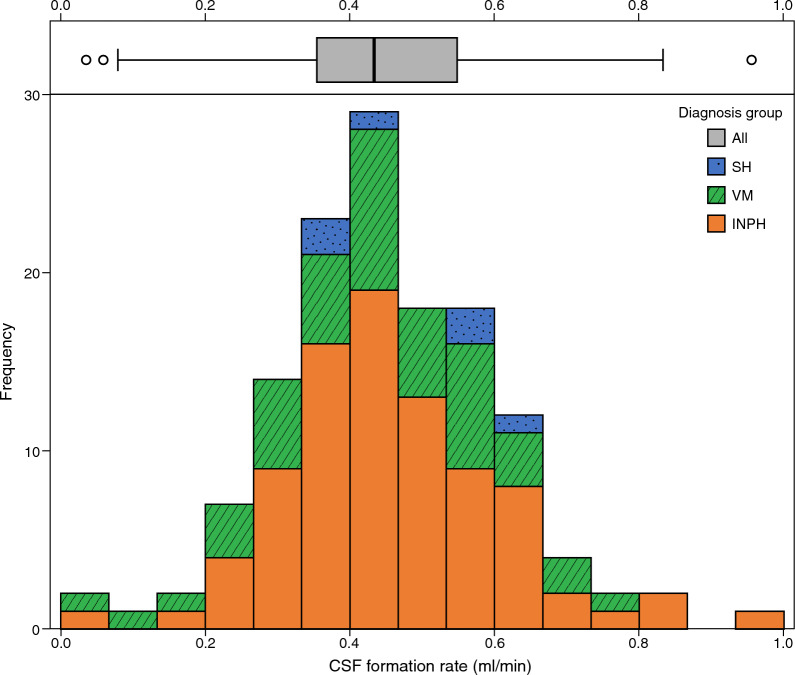
Boxplot and stacked histogram of CSF formation rate (Q_f_). The boxplot (top) shows all subjects together (vertical line: median, box: first and third quartile, bar: range excluding outliers; circles: statistical outliers). The stacked histogram (bottom) displays the different diagnoses (INPH: orange solid, VM: green dashed, SH: blue dotted area), stacked on top of each other such that the full height of the bars illustrates the histogram for the entire cohort

For the entire cohort, estimated Q_f_ was 0.45 ± 0.15 ml/min (mean ± SD, N = 135), equivalent to 27 ± 9 ml/hour; the distribution is shown in Fig. 2. There was no difference in average Q_f_ in subjects with INPH and VM (0.46 ± 0.15 ml/min, N = 86 vs. 0.43 ± 0.16 ml/min, N = 43; p = 0.362) or in the variance of CSF formation rate (p = 0.498). Average Q_f_ in SH was 0.49 ± 0.11 ml/min. The group of patients with INPH (N = 86) had slightly higher Q_f_ than the reference cohort (0.46 ± 0.15 vs. 0.40 ± 0.08 ml/min, p = 0.005), and a larger standard deviation (p < 0.001).

Q_f_ was 0.47 ± 0.13 ml/min for men and 0.43 ± 0.17 ml/min for women (N = 64 vs. 71, all diagnoses); there was no statistically significant difference in mean (p = 0.180) or variance (p = 0.375) between these groups.

In the uncertainty analysis the standard error of the Q_f_ estimates showed a median of 0.09 ml/min and interquartile range 0.05–0.16 ml/min for the whole cohort. The corresponding values for the INPH group were median 0.09 ml/min and interquartile range 0.05–0.17 ml/min.

**Fig. 3:**
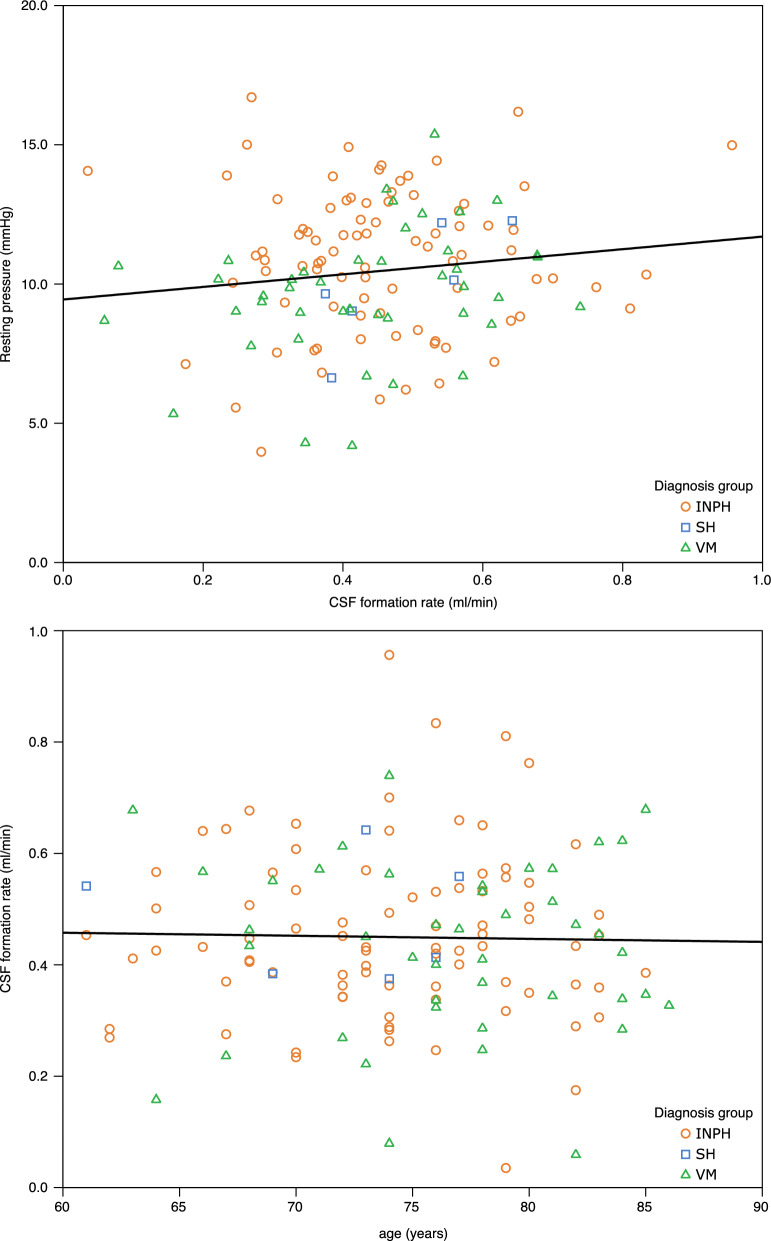
Scatter plots of resting pressure vs. CSF formation rate (top panel) and CSF formation rate vs. age (bottom panel). The black lines represent the linear regression corresponding to the correlations for the entire cohort; colors and symbols correspond to which diagnosis group an individual belongs to

There were no correlations between Q_f_ and P_baseline_ (r = 0.136, p = 0.115) or age (-0.02, p = 0.803, N = 135); these relationships are illustrated in Fig. 3. There was a trend towards correlation between P_baseline_ and age (r = − 0.164, p = 0.058, N = 135), but correcting for this relationship had negligible effect on the correlation between Q_f_ and P_baseline_ (partial correlation coefficient: 0.135, p = 0.121) or between Q_f_ and age (partial correlation coefficient: 0.01, p = 0.994). Analyzing only the patients with INPH did not result in stronger correlation between Q_f_ and P_baseline_ (r = 0.032, p = 0.773, N = 86) or Q_f_ and age (r = 0.01, p = 0.924).

## Discussion

We here present the CSF formation rate of a large patient cohort with various forms of hydrocephalus and compare the values for patients with INPH to those of a historical reference cohort [11]. The formation rate was measured with an established method that provides reliable results on the group level. The measured Q_f_ in INPH was statistically higher than in the reference cohort (15% higher in INPH), but this slight difference was probably not physiologically relevant. There was no difference between subjects with different verified diagnoses (INPH/SH/VM). Thus, our findings do not support the previous finding that Q_f_ is reduced in chronic hydrocephalus [6]. They also indicate that reduced CSF turnover in INPH, as previously demonstrated by glymphatic MRI [2–5], is not a result of a reduction in CSF formation rate. Further research is needed to clarify the mechanism behind impaired glymphatic flow in INPH.

There are two potential mechanisms by which CSF formation rate may play a role for clearance, depending on the glymphatic efflux pathways, i.e., if glymphatic efflux from the brain passes directly to venous and/or lymphatic drainage or if CSF recirculates to the subarachnoid space after passing through the glymphatic system [18]. In the former case, the CSF formation rate will directly limit how much CSF flow that is available for glymphatic clearance, since formation and efflux/absorption must be balanced to maintain equilibrium of the CSF volume. In the latter case, if there is mixing of the CSF flowing into and out of the glymphatic system, the flow rate in the glymphatic system is not dependent on the formation of CSF, but the formation rate will still affect how quickly the CSF is “refreshed” with metabolite-free fluid. Glymphatic MRI after intrathecal tracer injection in INPH has shown evidence of slower clearance of CSF tracer in the parenchyma [2, 3], suggesting slower CSF flow in the glymphatic system, while blood samples from patients undergoing the procedure suggest delayed clearance of tracer from CSF to blood [4]. Together, these findings have been interpreted as reduced CSF turnover. Our findings, that CSF formation rate in INPH is more likely to be increased than reduced, does not support this interpretation for the overall CSF circulation, since this turnover is a function of the formation rate. Normal (or increased) CSF formation rate in combination with slower flow of CSF in the glymphatic system in INPH could be the result of a smaller proportion of the CSF flow passing through the glymphatic system, but it also highlights the possibility of CSF recirculation to the subarachnoid space. The delayed clearance of tracer from CSF to blood in INPH could seem contradictory to our findings but may be a result of a redistribution between different types of absorption routes for the CSF, with increased efflux to lymphatic drainage, causing a delayed route to blood, as opposed to directly to blood. An important aspect of the method used in this study is that it measures the total replenishment of CSF volume, regardless of where the CSF is formed. According to the traditional concept of CSF circulation, CSF is produced by the choroid plexus, but it has been suggested that the capillaries may be an additional source of CSF. Potentially, the relative contributions of different sources of CSF formation could also relate to the effectiveness of glymphatic clearance, and this cannot be evaluated based on our results. Further research in humans is needed to elucidate the glymphatic flow pathways and thus also the more exact role of the CSF formation rate.

The distribution of the assessed Q_f_ rate was wide, with values in some subjects as low as half, or as high as twice the average value (see Fig. 2). The variance in our study was wider than in the reference cohort, but while this may relate to the pathology of the patients it is likely that it largely results from the longer measurement interval in the study by Eksted (20–30 min vs 10 min) [11]. Any variability in the pressure during the measurement will lead to uncertainties in the estimation of Q_f_. Physiological variability in pressure will typically not be related to changes in Q_f_, but to, e.g., changes in cerebral blood volume, and thus the corresponding changes in pump flow will reflect noise in the Q_f_ estimate. A longer measurement period should produce a more stable average value, as this noise is averaged out. This is supported by our uncertainty analysis, where the median standard error for the 10-min measurements was 0.09 ml/min, which is a substantial part of the standard deviation of the cohort (0.15 ml/min). For an estimate based on twice the time (20 min), the uncertainty should theoretically scale as 1/√2. This could thus explain most of the difference in standard deviation between the cohorts. This motivates that future measurements to determine CSF formation rate on an individual basis should measure for at least 20 min, and determine that the standard error is at an acceptable level.

Table 2 presents previous publications on invasive measurements of CSF formation rate in humans, with either healthy subjects or hydrocephalus patients. Several methods have been suggested [19], but only invasive methods can claim to measure the total formation rate in humans. Thus, we here limit the presented studies to methods based on withdrawal of CSF.

**Table 2:** Summary of studies with invasive measurement of CSF formation rate in healthy and hydrocephalus

Author, Year	N	Subjects	Age [years]	Formation rate [ml/mi]	Method & ICP interval
Masserman, 1931 [20]	42	Patients requiring LP incl. those with “no demonstrable disease” of the CNS	unknown	0.30	35 ml CSF withdrawal, ICP ≤ baseline
May et al., 1990 [8]	7 7	Healthy young Healthy older	(21–36) (67–84)	0.41 ± 0. 24 0.19 ± 0.07	10 ml CSF withdrawal, ICP ≤ baseline
Malm et al., 1995 [22]	35	INPH	(64 – 77)	0.5	ICP regulation ICP ~ 0.25 kPa
Silverberg et al., 2002 [6]	10	Chronic hydrocephalus	54 ± 24	0.25 ± 0.08	3 ml CSF withdrawal, ICP ≤ baseline
Edsbagge, et al. 2004 [21]	34	Healthy	25 (21–35)	0.34 ± 0.13	11–12.6 ml CSF withdrawal, ICP ≤ baseline
Tariq et al., 2023 [25]	41	Suspected NPH	77	1.32 ± 0.33	ICP regulation ICP ~ 0 kPa
Reference cohort: Ekstedt, 1978 [11]	58	Healthy (historical controls)	(15–85)	0.40 ± 0.08	ICP regulation ICP ~ 0.25 kPa
Present study	86 43 6	INPH VM SH	74 ± 6 77 ± 6 72 ± 6	0.46 ± 0.15 0.43 ± 0.16 0.49 ± 0.11	ICP regulation ICP ~ 0 kPa

Presented study populations are limited to healthy subjects and hydrocephalus patients. *NPH* normal pressure hydrocephalus, *LP* lumbar puncture, *INPH* Idiopathic NPH, *AD* Alzheimer’s disease, *PD* Parkinson’s disease

The methods that are based on bolus withdrawal of a certain volume of CSF, followed by monitoring of the time needed for return to the baseline pressure, have a major drawback in that the analysis does not take into account the natural CSF outflow. Specifically, some absorption would occur as soon as there is a pressure gradient to the lymphatic or dural venous pressure, and as ICP increases toward baseline absorption increases accordingly. This may explain why the values from these studies [6, 20, 21] are somewhat lower than those of the other studies in Table 2.

The studies by Ekstedt (the reference cohort) [11] and Malm et al. [22] used a very similar method to ours, but with CSF pressure regulated at 0.25 kPa (~ 2 mmHg), which is similar to the likely lymphatic pressure range of 0–2 mmHg [15, 16], so absorption to the lymphatic system should be negligible. This has relevance for the validity of the comparison between Ekstedt’s healthy cohort and our INPH group. There may be underestimation of Q_f_ in the Ekstedt study due to the pressure level being slightly above zero, which would not be present in our study. However, while it is difficult to predict the actual magnitude of this underestimation due to uncertainties in the lymphatic pressure as well as the contribution of this absorption route to the total CSF outflow absorption, it is likely to be small since the pressure gradient will be small and only present for absorption to the lymphatic system. Another aspect to consider here is whether Q_f_ is independent of CSF pressure in this range. This is supported by the current understanding of CSF formation as an active secretion process, that even can act against an osmotic gradient, rather than deriving from ultrafiltration of plasma [17, 23], as well as data suggesting that formation rate, as measured by ventricular perfusion, does not show any clear dependence on CSF pressure [24]. Altogether, we judge it unlikely that a pressure difference of 1.9 mmHg would alter our finding of slightly increased Q_f_ in INPH as compared to healthy, to a significant reduction in INPH, which was the hypothesis investigated. This is further supported by the average value for INPH patients in the Malm et al. study (0.5 ml/min), which used the same pressure level as Ekstedt, being larger than the average for the healthy cohort.

Tariq et al. [25] recently presented a large material with different pathologies, including a group with suspected NPH, where measurements were based on the same approach as our study but using a different device. They concluded that CSF formation rate may be unexpectedly high in several conditions, and indeed, the estimated formation rates in their study were substantially higher than all the previous studies for all but one of the investigated pathologies (pituitary adenomas) [25]. Their values for NPH are also substantially higher than ours, despite the seemingly similar groups and approaches, which raises the question of whether the increased rates they observed are related to pathology or to disparities between different devices and methodology.

It was somewhat surprising that there was no significant correlation between P_baseline_ and CSF formation rate (r = − 0.164, p = 0.058). According to the classical model based on the Davson equation, baseline CSF pressure will depend directly on formation rate [26, 27]. However, this relationship is also dependent on the resistance to CSF outflow and the dural sinus venous pressure, which both will have variability [13, 28], potentially leading to CSF formation rate´s low impact on the baseline pressure.

CSF formation is expected to decrease with age[8], which has been suggested as a possible contributor to reduced glymphatic clearance with aging[9]. This finding could not be reproduced in the present study. The study by May was based on 7 young and 7 old volunteers [8], making it very small compared to the present study; which has a substantially narrower age range (see Table 1). However, the study by Ekstedt et al. investigated a “healthy” cohort with a large age span, and also did not find a correlation between CSF formation rate and age [11]. Similar results were found by Silverberg et al. for a group with chronic hydrocephalus [6]. This supports the validity of the comparison between our study cohort and the Ekstedt cohort, despite the much wider age range in the older study (18–82 years vs. 61–81 years). From a glymphatic perspective it is interesting that the accumulation of beta-amyloid and tau has its onset long before AD-patients become symptomatic [29]. Therefore, a lifetime of low glymphatic flow, potentially related to a low CSF formation rate, may lead to accumulation that only causes symptoms as you get old.

## Conclusions

The average CSF formation rate in our cohort of 135 hydrocephalus patients was 27 ± 9 ml/hour. There was no difference between those with a diagnosis of INPH and those with other sub-diagnoses of hydrocephalus, and no correlation to age. Our results do not support a reduced overall CSF turnover as the reason for the impaired glymphatic clearance suggested in INPH, based on glymphatic MRI. These findings emphasize the need to further investigate the source and routes of the flow in the glymphatic system and the cause of the suggested impaired glymphatic clearance in INPH.

## Data Availability

The datasets analyzed during the current study are available from the corresponding author on reasonable request.

## Appendix

86 patients (64%) had an INPH diagnosis based on the international INPH guidelines [30].

6 patients (4%) had secondary hydrocephalus. Patients were classified as secondary hydrocephalus if there was a communicating hydrocephalus caused by subarachnoid hemorrhage, trauma, infection, or stroke.

43 patients (31%) were included in the group “ventriculomegaly”. This included patients with communicating hydrocephalus revealed on MRI, who did not meet the clinical criteria of the INPH guidelines, or those who had other neurodegenerative comorbidities (e.g. Parkinson's, motor neuron disease or stroke) in addition to an INPH diagnosis. Ventriculomegaly with other types of dementia (e.g., Alzheimer's, Binzwanger, vascular dementia) was also included in this group.

## Abbreviations

INPH: Idiopathic normal pressure hydrocephalus
CSF: Cerebrospinal fluid
Q_f_: CSF formation rate
P_baseline_: Baseline CSF pressure
SH: Secondary hydrocephalus
VM: Ventriculomegaly
SD: Standard deviation
